# Interspecific foraging response to the thiacloprid treatment of co-existing top spider predators

**DOI:** 10.1038/s41598-026-52307-y

**Published:** 2026-05-12

**Authors:** Anděla Šimečková, Filip Solar, Michaela Kolářová, Eva Líznarová, Stanislav Korenko

**Affiliations:** 1 Department of Agroecology and Crop Production, Faculty of Agrobiology, Food and Natural Resources, Czech University of Life Sciences Prague, Kamýcká 129, Suchdol, Praha, 165 00 Czech Republic; 2 Department of Botany and Zoology, Faculty of Science, Masaryk University, Kotlářská 2, Brno, 611 37 Czech Republic

**Keywords:** Ecology, Ecology, Zoology

## Abstract

Neonicotinoids are nicotine-based synthetic insecticides used in agriculture to control plant pests. They are neurotoxic substances that attack the nervous system of insects and can cause paralysis or death. These selective insecticides should have a negligible effect on non-target organisms, including spiders, which are one of the most abundant and diverse natural predators that contribute to the control of pests. Current studies show that selective insecticides such as neonicotinoids have negative effects on non-target organisms. They can have both lethal effects resulting in mortality, and sublethal effects involving various aspects of their lives, e.g. breeding, movement, hunting, ability to defend against predators, and predatory activity. We studied the species-specific responses to neonicotinoid treatment with the active ingredient thiacloprid of two top spider predators coexisting in tree crowns in Europe - respectively, spiders of the genus *Philodromus* (*aureoles* group, Philodromidae) and species *Anyphaena accentuata* (Walckenaer) (Anyphaenidae). Spiders were exposed to field-realistic concentrations of the tested substance, while the control group was treated with distilled water. We compared the species-specific responses of functional response and two components of predation rate: feeding and overkilling. Further, we monitored long-term survival and recovery from paralysis compared to control, and the impact of insecticide residues on predation rate 14 days post-exposure. We found that a one-hour tarsal contact with thiacloprid significantly reduced predation rate in both *Anyphaena* and *Philodromus* spiders, although the effects were highly species-specific. In *Anyphaena*, feeding was inhibited by fresh treatment, whereas *Philodromus* remained unaffected in this regard. Furthermore, the rate of overkilling significantly decreased under the fresh treatment in both species. The treatment induced a reversible paralysis in *Philodromus*, whereas it caused significant mortality in *Anyphaena*. Furthermore, 14 days post-treatment, the insecticide had no significant effect on feeding of either species, while the overkilling in *Philodromus* remained significantly lower than in the control. Overall, the study demonstrates a species-specific response to thiacloprid among top pest predators that share the same ecological niche in orchards.

## Introduction

Many pesticides and active substances that were considered safe when first introduced commercially were later found to have negative effects on certain groups of organisms^1–6^. These effects are often sublethal, meaning that they do not lead to immediate mortality, but may cause other harmful effects such as reduced fitness, changes in behaviour, or impaired development^7–13^. Such sublethal effects often go undetected during the registration process because standard testing focuses primarily on lethal effects^14^. As a result, the full range of ecological impacts, especially those occurring at sublethal levels, is often overlooked. This highlights the need for testing protocols that are more rigorous and comprehensive than EU Commission Regulation 2013 (No 283/2013) in order to account for such potential non-lethal impacts on non-target species. Although risk assessments have traditionally focused on a limited number of model species, recent large-scale evidence confirms that these negative impacts are broadly consistent across diverse taxonomic groups^6^.

Ever since neonicotinoids were commercialised in the 80s and widely used in agriculture to protect crops against insect pests, research has progressively revealed their significant negative impacts on various important groups of organisms^15,16^. For example, studies have linked neonicotinoid exposure to widespread losses of honeybee colonies, raising concerns about their wider ecological consequences^17–19^. These insecticides have been shown to affect not only pollinators but also other non-target organisms, including beneficial arthropods, aquatic invertebrates, and birds, leading to declines in populations over large areas^20–23^. Spiders are generally less sensitive to neonicotinoids than insects due to differences in the structure of their acetylcholine receptors^24^. Nevertheless, several studies have documented significant sublethal effects on spider physiology and behavior. These include temporary paralysis^25^, changes in silk production^26^, disruption of chemoreception^27^, and a decrease in predation rate^25^. All these together cause a significant reduction in spiders’ potential for biological pest control and can damage their local populations in agroecosystems.

There are restrictions on neonicotinoid use to mitigate their adverse effects on ecosystems. For example, most neonicotinoid active substances have been banned in the European Union since 2020, except for acetamiprid, which is considered to be less of a risk to bees^28^. However, these substances are still actively used in various parts of the world, including Latin America, Asia and North America^29^.

Pesticide sensitivity can vary significantly even among ecologically similar species. For example, Jütte et al.^30^ exposed seven bee species, including *Apis mellifera* (Linnaeus 1758), *Bombus terrestris* (Linnaeus 1758) and several solitary species, to field‑realistic levels of the pyrethroid insecticide lambda‑cyhalothrin. They observed marked species-specific differences in both mortality and sublethal behavioral effects. This challenges the adequacy of using *A. mellifera* as a sole surrogate in risk assessments. Similarly, Short et al.^31^ found over 30-fold differences in imidacloprid sensitivity among five earthworm species, explained by the species-specific expression of nonclassical acetylcholine-binding proteins. Henriques Martins et al.^32^ further demonstrated that different dipteran pollinators exhibit species-specific sensitivity to imidacloprid, with LD₅₀ values varying by nearly twofold. The authors also showed that sublethal endpoints, such as fecundity, can be highly sensitive indicators of pesticide effects. While specific responses to neonicotinoids including thiacloprid have already been documented in spiders by^25^, previous research focused on species from three different foraging guilds and microhabitats - respectively, *Pardosa lugubris* (Walckenaer 1802) (Lycosidae), a dominant ground dwelling spider; *Philodromus cespitum* (Walckenaer 1802) (Philodromidae), inhabiting tree crowns; and sheet-weaving spiders from Linyphiidae inhabiting vegetation. These interspecific differences in pesticide sensitivity were expected because of differences in ecology and microhabitat adaptations. To accurately assess how pesticides affect pest control, we must understand their impact on a broad range of predator species within the habitat, rather than focusing on a single taxon.

The main objective of this research was to evaluate the species-specific impact of thiacloprid on the functional response, predation rate, mortality and occurrence of paralysis of co-existing top predators. We focused on two dominant spider species from the same foraging guild that share the same microhabitat and play a similar role in the biological control of pests in orchards. As models we used two common tree crown and shrub dwelling spiders - *Anyphaena accentuata* (Walckenaer 1802) (Anyphaenidae) and *Philodromus* spp. (*aureolus* group, Philodromidae). Both species are top predators of insect pests in European orchards^33^ and their ecological niches and prey spectrum overlap considerably^34^. Both are known as winter-active spiders, which are important in reducing hibernating insect pests during the winter season^35,36^. Despite these similarities, these two species differ in their temporal activity pattern. *Anyphaena* is a nocturnal hunter that effectively preys on non-flying aphids and lepidopteran larvae, with recent studies confirming a strong preference for hemipterans, especially pear psyllids, during winter^34,37,38^. In contrast, *Philodromus* spiders are diurnal hunters of hymenopterans and lepidopterans^37^, but also frequently prey on other spiders, dipterans, and hemipterans, indicating a broad and opportunistic diet^34^. Spiders regulate prey populations not only through direct consumption but also by overkilling - a behavior in which individuals kill more prey than they consume^39^. This phenomenon is observed across various species, particularly in active hunters and its occurrence typically increases with prey density^40,41^. While overkilling may represent an adaptive strategy to avoid ingesting unsuitable or toxic prey^42^, an alternative interpretation suggests it is a non-adaptive by-product of increased predatory aggression^43,44^.

In the present study we focused on thiacloprid, an active substance belonging to the chloronicotinoid group. This compound acts as a contact and ingestive poison with systematic effects, primarily inhibiting impulse transmission within the insect nervous system. Although its mechanism of effect is similar to that of acetylcholinesterase inhibitors, but thiacloprid is inactivated only slowly. This persistence leads to general nervous system dysfunction and subsequently to the death of the affected target organism^45^. Commercially, thiacloprid serves as the active ingredient in several formulations, including Biscaya, Calypso, Proteus, and Sonido (Bayer CropScience).

We investigated the effect of thiacloprid on the predation rate of two coexisting top predators, *Anyphaena* and *Philodromus* spiders. Specifically, we evaluated the functional response as a response of predator to density of prey and predation rate, which included feeding and overkilling^41^. The spiders were treated only once, with their predation rate and functional response measured both immediately after the treatment. 14 days post-exposure we measured the predation rate again to capture the residual effects. Post-treatment monitoring lasted four weeks for mortality and two weeks for paralysis.

We tested the hypothesis that treatment with the active ingredient thiacloprid negatively affects the traits of the functional response and predation rate of both *Anyphaena* and *Philodromus* spiders. Furthermore, we hypothesized that exposure would induce neurotoxic effects, specifically mortality and paralysis. To evaluate the persistence of these impacts, we also monitored predation rate 14 days post-treatment. Finally, we expected that these two co-existing top spider predators would exhibit different sensitivities to both the lethal and sublethal impacts of the pesticide, potentially leading to varied consequences for their biocontrol services.

## Materials and methods

### Spider collection and laboratory rearing

Individuals of two coexisting top predators, *Anyphaena accentuata* (Anyphaenidae) and *Philodromus* spp. (Philodromidae; *aureolus*-group, with > 70% dominance of *P. cespitum*), were collected from apple orchards and their surroundings in Prague–Sedlec, Czech Republic (50.13°N, 14.39°E) during September and October 2023.

The spiders were kept individually in 10 ml plastic tubes (diameter 15 mm; length 50 mm) with pierced lids to ensure ventilation. A layer of gypsum plaster at the bottom was moistened weekly with distilled water to maintain humidity. All individuals were kept under laboratory conditions with natural photoperiod at temperature of 19 ± 3 °C, relative humidity 60 ± 10%. The spiders were fed to satiation once a week with wingless *Drosophila melanogaster* (Meigen, 1830) cultivated on a commercial NEKTON medium. To standardize hunger levels and ensure high foraging motivation, spiders underwent a 14-day starvation period prior to the treatment.

### Insecticide application and exposure

The experiments were conducted under standard laboratory conditions at the Department of Agroecology and Plant Production, Czech University of Life Sciences Prague (Prague, Czech Republic). Treatment and subsequent predation rate testing were performed at room temperature of 20 ± 2 °C during the day. We used neonicotinoid insecticide with the active substance thiacloprid which was formulated as Biscaya 240 OD (Bayer CropScience, Monheim, Germany). The insecticide and distilled water as a control were applied on filter paper sheets at the manufacturer’s recommended dilution for crop spraying (thiacloprid concentration 240 g/L, 23.1%), with a suggested application rate of 250 ml ha⁻¹. Filter paper sheets were treated with 0.737 ml of the solution (or distilled water for the control) using an automatic pipette. The entire surface was uniformly moistened and the sheets were left to dry for 30 min. Afterwards the sheets were rolled into tubes (diameter 15 mm; length 50 mm) covering the entire inner surface to ensure continuous tarsal contact. Each spider was transferred individually into the treated tubes for a 60-minute exposure period. The spiders were treated only once by tarsal contact.

### Effect on functional response and predation rate immediately after the treatment

To evaluate the immediate effect (fresh treatment) of thiacloprid, the functional response was assessed as total predation rate, incorporating all captured prey. Both feeding (prey consumed) and overkilling (prey killed but not consumed) behaviors were recorded, with their frequency expressed as a function of prey density. Spiders were transferred individually to Petri dishes (height 1 cm; diameter 5.5 cm) right after the 60-minute exposure period and allowed a 15-minute acclimatization period. During this time, each spider’s body length was measured using millimeter paper, placed underneath each Petri dish. We tested a total of 150 *Philodromus* spp. and 150 *A. accentuata* individuals. Within each species, half of the individuals (*n* = 75) were treated with the insecticide, while the other half served as a control (distilled water).

To determine the functional response and predation rate spiders were divided into groups based on the number of offered prey: 1, 3, 6, 9, 12 (15 spiders in each group). There were no significant differences in body length between the control groups and the treated *Anyphaena* (Wilcoxon test, W = 2738, *p* = 0.8867) and *Philodromus* groups (Wilcoxon test, W = 2741.5, *p* = 0.2562). Living wingless *D. melanogaster* were provided as a prey and were maintained at constant densities according to the number of each group. Captured prey was replaced every 30 min for 4 h (total of 8 rounds). During each round, two prey capture behaviors were recorded: feeding (at least 30% of the prey body consumed) and overkilling (more than 70% of the prey remained).

### Effect on predation rate 14 days post-exposure

To assess potential recovery or residual effects, predation rate was re-evaluated 14 days post-exposure (referred to as residues in the figures).This interval was chosen to determine if the immediate effects of the treatment would diminish over time. The 14-day interval reflects the environmental persistence of the substance, as European field dissipation studies report a DT50​ for thiacloprid ranging from 6 to 17 days^46^. Spiders were not fed during this 14-day period, as they had been allowed to feed to satiation during the initial testing. Individuals that exhibited highest functional response and predation rate during the immediate post-treatment testing were chosen for assessing the effect after 14 days post exposure. A total of 30 *Anyphaena* and 30 *Philodromus* spiders (15 treated and 15 control individuals per species) were tested for functional response and predation rate again. Based on the previous results a constant number of 6 prey was used, as predation rate was generally highest at this density. The experimental design followed similar protocol as the prior testing: a constant density of 6 *D. melanogaster* was maintained for 4 h (8 rounds), with all captured prey replaced every 30 min. The same two prey capture behaviors were recorded: feeding (at least 30% of the prey body consumed) and overkilling (more than 70% of the prey remained).

### Mortality and paralysis

Additionally, we monitored spider mortality and visible signs of paralysis both during the experiment and at specific times following thiacloprid exposure. Mortality was recorded immediately post-exposure and subsequently at 1, 2, 3 and 4 weeks to assess delayed effects. Paralysis was defined as a marked reduction in mobility where spiders appeared unusually limp, but without the characteristic stiff and curled posture of dead individuals. Paralysis was recorded at 1 and 2 weeks post-exposure. The paralysis was further characterized by its reversibility, accordingly paralysis was monitored for a period of 14 days, as all individuals had recovered normal locomotor activity during this period.

### Statistical analyses

Predation rate in response to prey density (number of killed prey, overkilling rate, and consumption rate) was analyzed using a generalized linear model (GLM) with Poisson distribution. These data fitted with a Type II functional response curve following the Holling^47^ disc equation. To assess whether the treatment effect differed between species, we used post hoc pairwise interaction contrasts based on the GLM using the emmeans package (estimated marginal means). The residual effect of thiacloprid on predatory activity 14 days post-exposure was tested using a GLM with Poisson distribution. We used a generalized linear model (GLM) with binomial distribution to test the effect of treatment on spider survival rate. We performed all statistical analyses in the R environment^48^.

## Results

### Species specific response to thiacloprid treatment

**Fig. 1:**
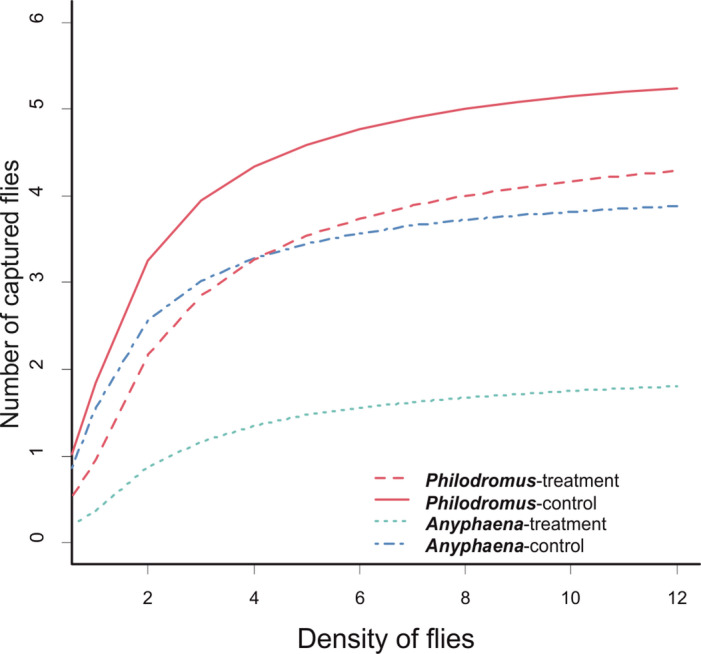
Functional response of *Philodromus* (GLM-p, χ^2^_1_ = 4.534, *p* = 0.033) and *Anyphaena* (GLM-p, χ^2^_1_ = 59.137, *p* < 0.001) spiders following fresh thiacloprid treatment compared to the control groups and between species (interaction contrast = 0.88, z = 7.15, *p* < 0.0001).

The functional response (predation rate response to prey density) of untreated spiders was significantly higher in *Philodromus* than in *Anyphaena* spiders (GLM-p, χ^2^_1_ = 10.535, *p* = 0.001, Fig. 1). The fresh treatment with thiacloprid significantly reduced the functional response in both *Philodromus* (GLM-p, χ^2^_1_ = 4.534, *p* = 0.033, Fig. 1) and *Anyphaena* (GLM-p, χ^2^_1_ = 59.137, *p* < 0.001, Fig. 1). This reduction was significantly stronger in *Anyphaena* compared to *Philodromus* (interaction contrast = 0.88, z = 7.15, *p* < 0.0001, Fig. 1).

**Fig. 2:**
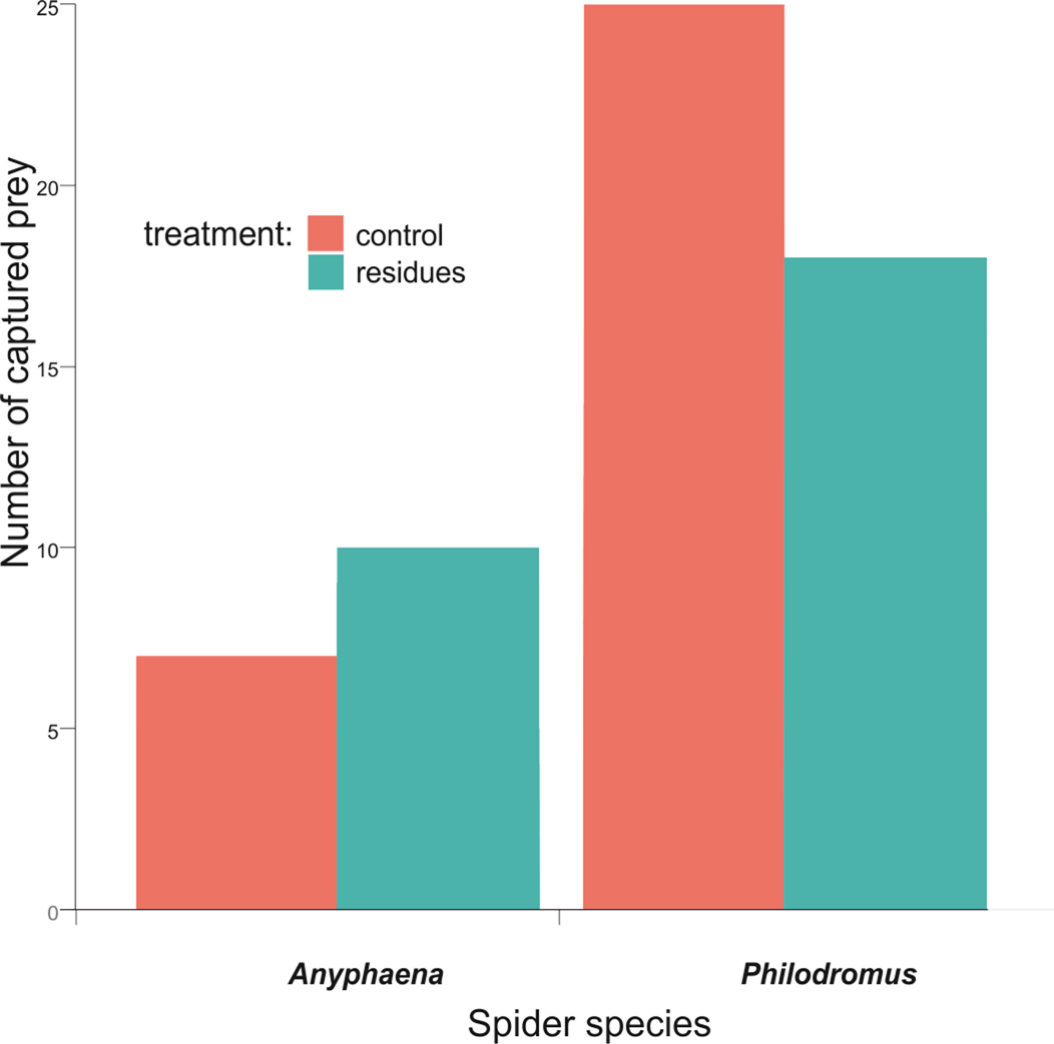
Residual effect of thiacloprid on the predation rate (number of killed flies) of *Philodromus* (GLM-p, χ^2^_1_ = 6.083 *p* = 0.014) and *Anyphaena* (GLM-p, χ^2^_1_ = 0.22, *p* = 0.633) spiders 14 days post-exposure compared to the control groups and between species (GLM-p, χ^2^_1_ = 154.703, *p* < 0.001).

The predation rate (number of captured prey) of spiders 14 days post-exposure differed significantly between *Philodromus* and *Anyphaena* spiders. Whereas *Philodromus* spiders exhibited a higher predation rate than *Anyphaena* spiders (GLM-p, χ^2^_1_ = 154.703, *p* < 0.001, Fig. 2). At 14 days post-exposure (residual effect), the number of flies killed by *Anyphaena* spiders did not significantly differ from the control group (GLM-p, χ^2^_1_ = 0.22, *p* = 0.633, Fig. 2). In contrast, *Philodromus* spiders 14 days post-exposure still killed significantly fewer flies than the control group (GLM-p, χ^2^_1_ = 6.083 *p* = 0.014, Fig. 2).

**Fig. 3:**
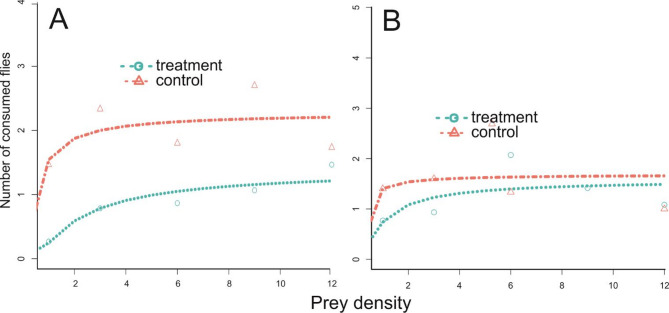
Feeding of *Anyphaena* (**A**); (GLM-p, χ^2^_1_ = 34.564, *p* < 0.001) and *Philodromus* (**B**); (GLM-p, χ^2^_1_ = 1.0601, *p* = 0.303) spiders following fresh thiacloprid treatment compared to the control groups at different prey density.

The effect of thiacloprid on feeding and overkilling differed significantly between *Philodromus* and *Anyphaena* spiders (Table 1). The feeding (number of consumed prey) of *Anyphaena* significantly decreased following the fresh treatment (GLM-p, χ^2^_1_ = 34.564, *p* < 0.001, Fig. 3A). In contrast, the feeding of *Philodromus* did not decrease significantly following fresh thiacloprid treatment (GLM-p, χ^2^_1_ = 1.0601, *p* = 0.303, Fig. 3B).

**Table 1:** Response of different traits to thiacloprid treatment in *Philodromus* and *Anyphaena* spiders in fresh and 14 days post-experiment. “–” means no data collected.

Trait	Time Period	Anyphaena accentuata	Philodromus spp.
Functional response	Fresh	↓ χ^2^_1_​=59.14,*p* < 0.001	↓ χ^2^_1_ = 4.53,*p* = 0.033
	14 days post-exp.	–	–
Predation rate	Fresh	↓ (Included in Func. Resp.)	↓ (Included in Func. Resp.)
	14 days post-exp.	No effect (χ^2^_1_​=0.22,*p* = 0.633)	↓ (χ^2^_1_​=6.08,*p* = 0.014)
Feeding	Fresh	↓ (χ^2^_1_​=34.56,*p* < 0.001)	No effect (χ^2^_1_​=1.06,*p* = 0.303)
	14 days post-exp.	No effect (χ^2^_1_​=2.79,*p* = 0.095)	No effect (χ^2^_1_​=1.01,*p* = 0.315)
Overkilling	Fresh	↓ (χ^2^_1_ = 25.18,*p* < 0.001)	↓ (χ^2^_1_​=3.74,*p* = 0.016)
	14 days post-exp.	No effect (χ^2^_1_​=0.52,*p* = 0.472)	↓ (χ^2^_1_​=5.90,*p* = 0.015)
Mortality	Fresh	↑ (χ^2^_1_​=36.24,*p* < 0.001)	No effect (χ^2^_1_​=1.89,*p* = 0.170)
Paralysis	Fresh	No	Yes (χ^2^_1_​=40.20,*p* < 0.001)

**Fig. 4:**
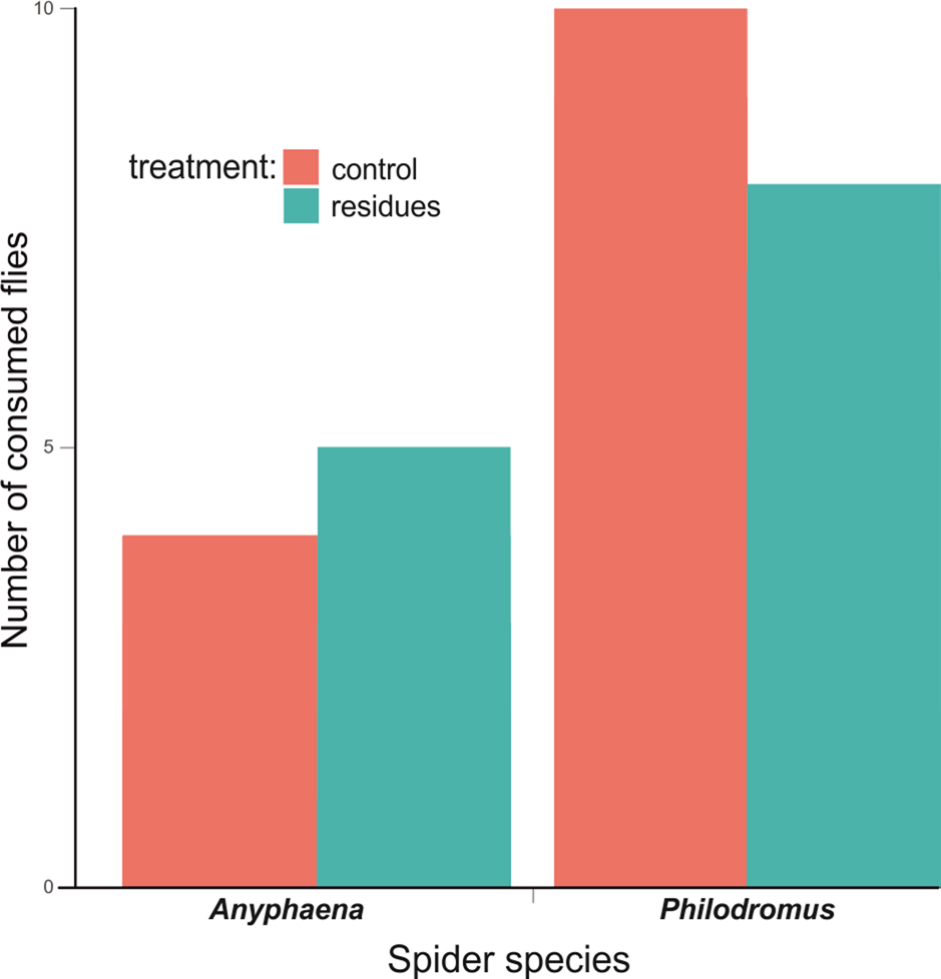
Residual effect of thiacloprid on feeding (number of consumed flies) of *Philodromus* (GLM-p, χ^2^_1_ = 154.703, *p* < 0.001) and *Anyphaena* spiders 14 days post-exposure compared to the control groups and between species (GLM-p, χ^2^_1_ = 154.703, *p* < 0.001).

The feeding (number of consumed prey) of spiders 14 days post-exposure differed significantly between *Anyphaena* and *Philodromus* spiders; the feeding rate was overall higher in *Philodromus* (GLM-p, χ^2^_1_ = 154.703, *p* < 0.001, Fig. 4). The feeding rate did not differ significantly between 14 days post-exposure and the control group in either *Anyphaena* (GLM-p, χ^2^_1_ = 2.789, *p* = 0.095, Fig. 4) or *Philodromus* (GLM-p, χ^2^_1_ = 1.0085, *p* = 0.315, Fig. 4).

**Fig. 5:**
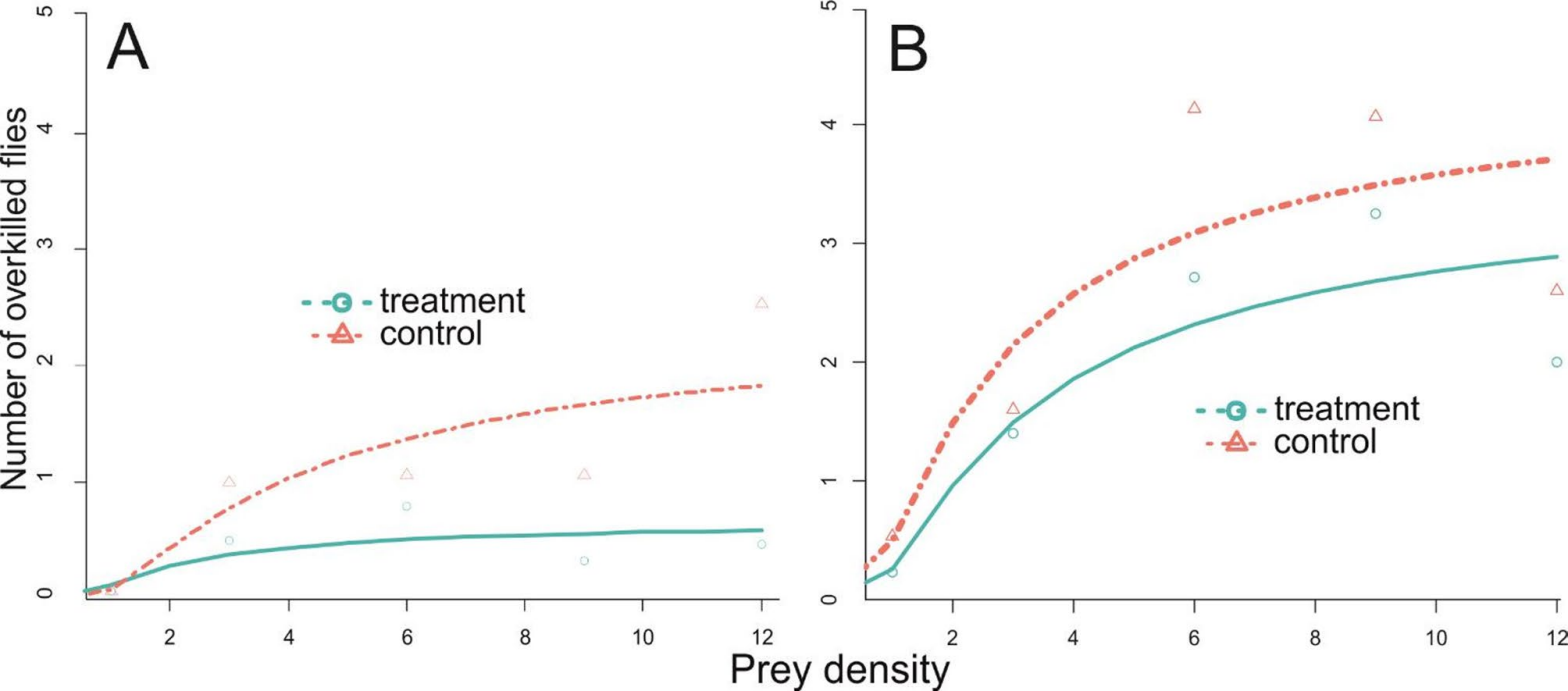
Overkilling rate of fresh-thiacloprid-treated *Anyphaena* (**A**); (GLM-p, χ^2^_1_ = 25.1767, *p* < 0.001) and *Philodromus* (**B**); (GLM-p, χ^2^_1_ = 3.741, *p* = 0.016) spiders compared to the control group at different prey density. Points show the average number of eaten prey at each density.

**Fig. 6:**
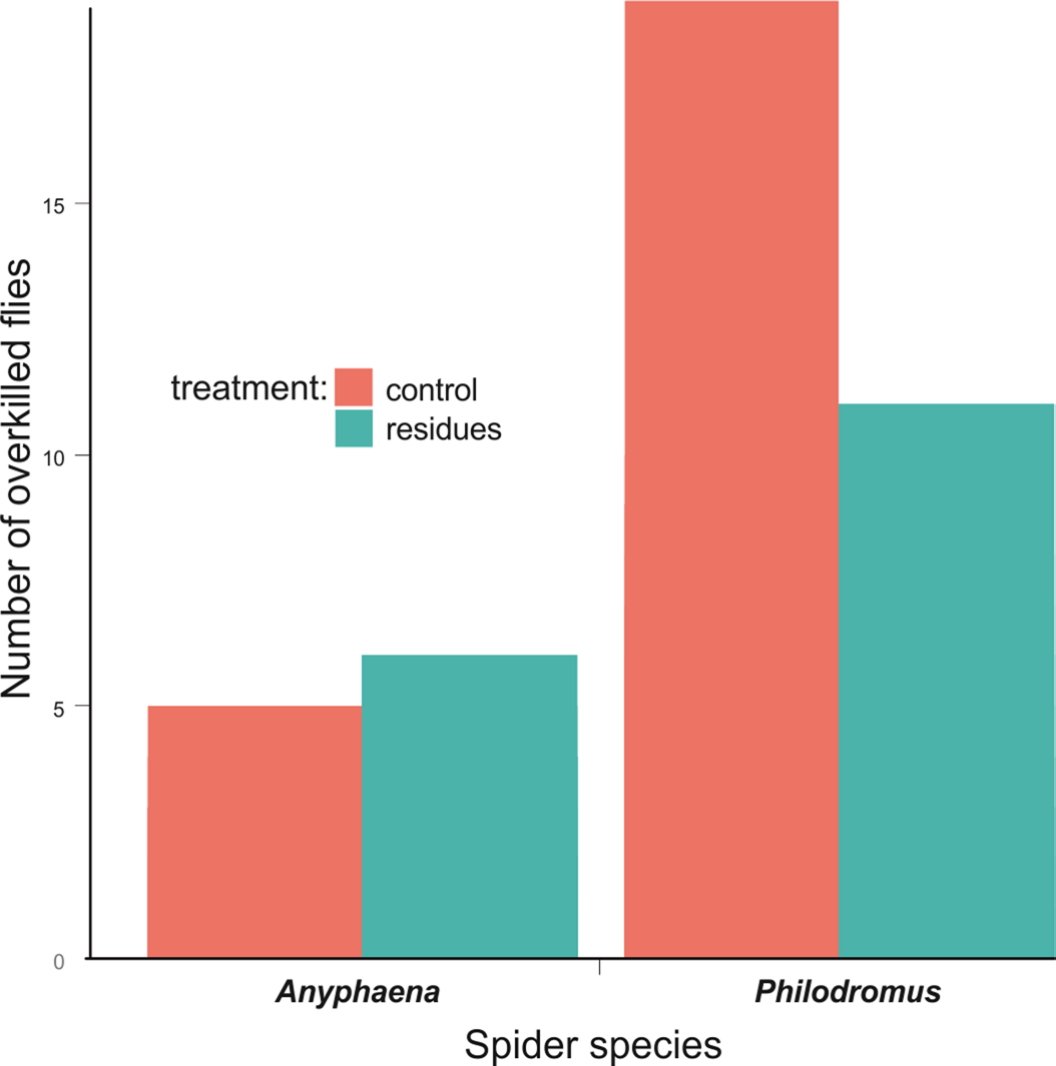
Residual effect of thiacloprid on overkilling (number of overkilled flies) of *Anyphaena* (GLM-p, χ^2^_1_ = 0.516, *p* = 0.472) and *Philodromus* (GLM-p, χ^2^_1_ = 5.899, *p* = 0.015) spiders 14 days post-exposure compared to the control groups and between species (GLM-p, χ^2^_1_ = 75.314, *p* < 0.001).

The fresh treatment significantly reduced the overkilling rate in both species compared to the controls; in *Anyphaena* (GLM-p, χ^2^_1_ = 25.1767, *p* < 0.001, Fig. 5A) and in *Philodromus* (GLM-p, χ^2^_1_ = 3.741, *p* = 0.016, Fig. 5B). The rate of overkilling by spiders 14 days post-treatment was significantly higher in *Philodromus* spiders than in *Anyphaena* (GLM-p, χ^2^_1_ = 75.314, *p* < 0.001, Fig. 6). The overkilling rate of *Anyphaena* did not differ significantly between 14 days post-exposure and control (GLM-p, χ^2^_1_ = 0.516, *p* = 0.472, Fig. 6), but it was significantly lower in *Philodromus* spiders 14 days post-exposure than in the control (GLM-p, χ^2^_1_ = 5.899, *p* = 0.015, Fig. 6).

### Mortality

**Fig. 7:**
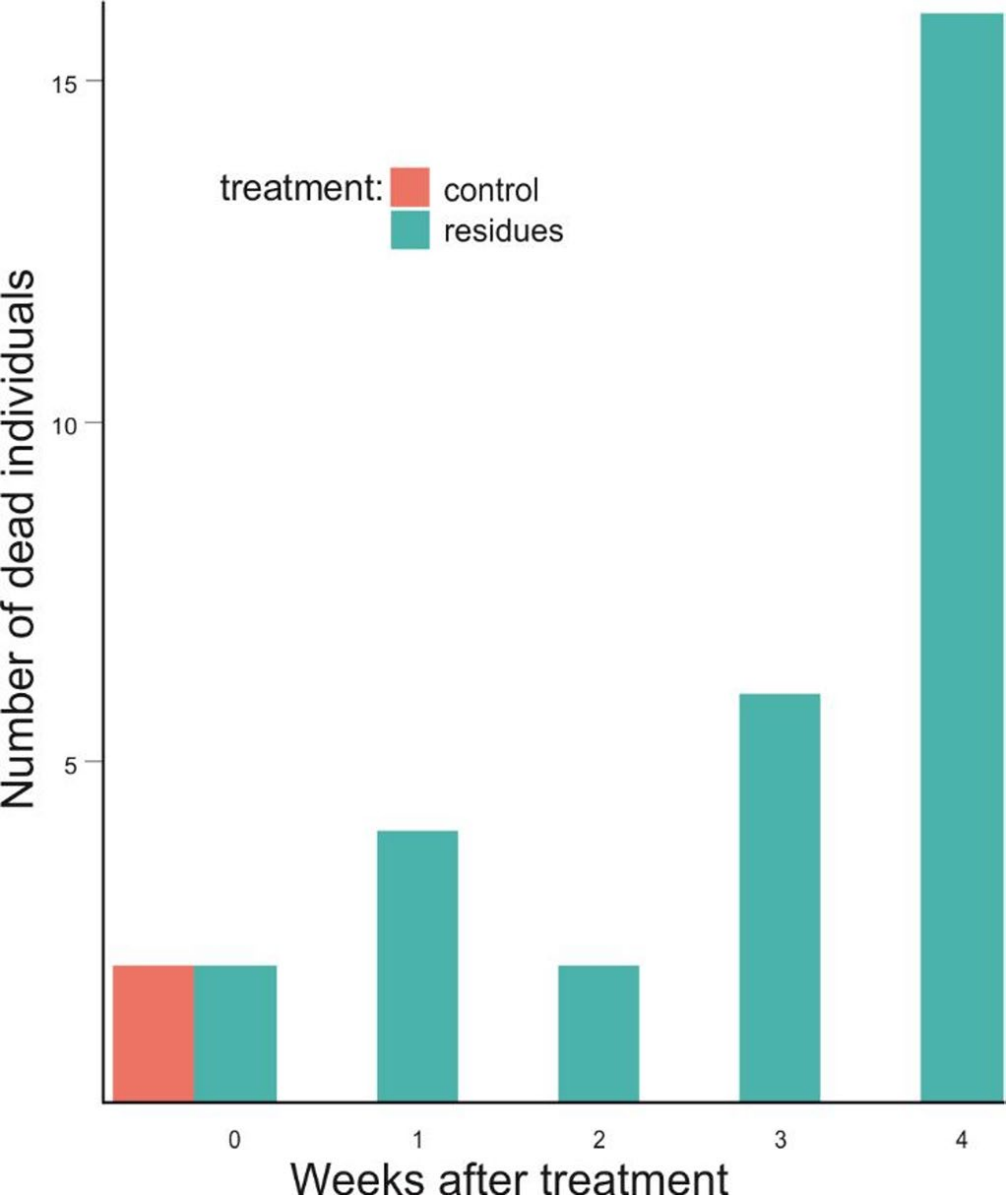
Mortality over time of *Anyphaena* spiders during the four-week post-exposure period following fresh thiacloprid treatment compared to the control (GLM-b, χ^2^_1_ = 36.238, *p* < 0.001).

The fresh treatment with thiacloprid did not cause significant mortality in *Philodromus* spiders (GLM-b, χ^2^_1_ = 1.887, *p* = 0.1695). In contrast, mortality in *Anyphaena* spiders was significantly higher in treated group compared to the control (GLM-b, χ^2^_1_ = 36.238, *p* < 0.001, Fig. 7). Following fresh thiacloprid treatment, 30 of the 75 *Anyphaena* spiders died (40%), whereas only two individuals died in the control group 2.7% of the total number. Of the total mortality in treated *Anyphaena* group, 7% occurred immediately after treatment, 13% within one week, 7% within two weeks, 20% within three weeks and the majority (53%) within four weeks (Fig. 7). Both individuals (100%) that died in the control group died within the first week.

### Paralysis

**Fig. 8:**
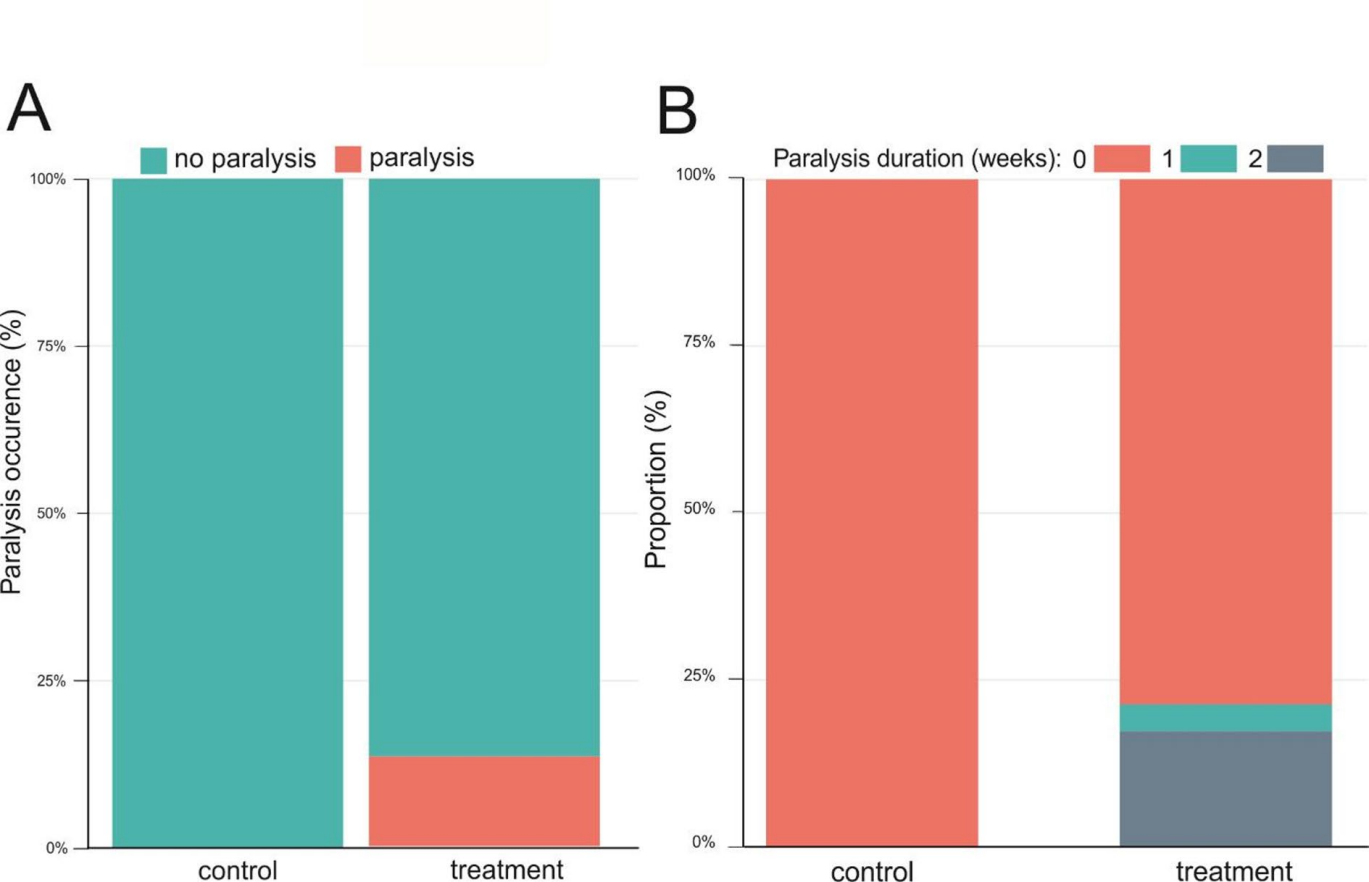
(**A**) Occurrence of paralysis (GLM-b, χ^2^_1_ = 40.203, *p* < 0.001) and (**B**) duration of paralysis in *Philodromus* spiders following fresh thiacloprid treatment compared to the control.

The fresh treatment with thiacloprid induced paralysis in 13% of *Philodromus* spiders, whereas no symptoms of paralysis were observed in the control group (GLM-b, χ^2^_1_ = 40.203, *p* < 0.001, Fig. 8A). Paralysis occurred immediately following the exposure of shortly afterwards. Spiders exhibited time dependent recovery, one week after treatment, 19% of the paralyzed spiders regained their mobility, and two weeks post-exposure, 100% of the paralyzed individuals had fully recovered (Fig. 8B). In contrast, no symptoms of reversible paralysis were observed in *Anyphaena* spiders following the treatment.

## Discussion

### Predation

The ability of predators to suppress pests is determined by the rate of predation on pests, which varies depending on prey density and can be expressed as a functional response^47^. A high increase in the number of prey is typical when the pest population is overpopulated, and therefore a positive functional response of the predator is a strong predictor of its effectiveness in crop pest suppression. Both studied species, *Anyphaena* and *Philodromus*, are dominant active predators on fruit trees^33^. Our results confirm a strong functional response already observed by Řezáč et al.^49^ in *Philodromus* and also revealed it in *Anyphaena* spiders for the first time. The absolute number of prey caught under laboratory conditions does not fully reflect the situation in nature because a saturated predator may avoid prey, or prey may escape. Predation measured in the laboratory differs from predation in nature, but laboratory data can approximate our knowledge of the situation in nature and indicate the predatory potential of the studied species. Such ecotoxicological laboratory data are valuable for assessing the effects of agrochemical treatments on predators.

### Effect of thiacloprid treatment

We report for the first time a species-specific effect of neonicotinoid treatment with the active ingredient thiacloprid on the foraging ecology of two top spider predators co-occurring in tree crowns in Central Europe, respectively *Anyphaena* and *Philodromus* spiders. Both species belong to the same foraging guild of active hunters^50^ and represent some of the most important natural predators in fruit orchards, where they share the prey and habitat^36,51^ even during the winter^35,36^ and also engage in intraguild predation^52^.

In both hunter spiders, thiacloprid treatment significantly decreased predation rate at all tested prey densities. The functional response of treated spiders correlated with the functional response of the control group, but the predation rate was significantly lower in thiacloprid treatment groups. The predation rate of treated spiders decreased in both species at all prey densities: by more than half for *Anyphaena* and by more than a quarter for *Philodromus*. The greater negative effect of thiacloprid treatment on the predation rate of *Anyphaena* may be related to the physiological adaptation of nocturnal *Anyphaena* to lower temperatures than those experienced by diurnal *Philodromus*. At temperatures below freezing, the lower limit for predatory activity was set at − 3.73 °C for *Anyphaena* and − 1.2 °C for *Philodromus*. At increasing temperatures, the predatory activity of *Anyphaena* was highest at 15 °C and then declined. In contrast, the predation rate of *Philodromus* increased monotonically with temperature and reached a maximum at 30 °C, which was the highest temperature tested^35^. The link between differences in the effect of thiacloprid on the two spider hunters studied and the differences in their temperature adaptations is only a hypothesis and requires further research. However, it has already been documented that higher temperatures have a synergistic effect on the toxicity of thiacloprid, as observed in crayfish^53^.

A decrease in predation was also documented in other spiders, e.g. in *Pardosa pseudoannulata* (Araneae: Lycosidae) Widiarta et al.^54^. after imidacloprid exposure, and in *Pardosa agrestis* (Araneae: Lycosidae) after thiacloprid exposure^55^.

In addition, a reduction in predation after neonicotinoid treatment with thiacloprid was found by^25^, who also observed that dorsal application had a higher impact on spiders than tarsal application, the latter producing only moderate effects. In the present study, we found a significantly negative effect of tarsal exposure to thiacloprid on the predation rate of both *Anyphaena* and *Philodromus* spiders. Řezáč et al.^49^ also found that neonicotinoid (acetamiprid) reduced the predation due to prolonged prey handling. In contrast, no effect of neonicotinoids on feeding was observed in *Philodromus cespitum* (Araneae: Philodromidae) after exposure to acetamiprid^49^.

### Predation, feeding, overkilling

The predation rate expresses the number of prey caught, which includes two different components: feeding, i.e., prey caught and completely or partially consumed, and so-called overkilling, i.e., prey caught but not consumed by the predator^41^. Feeding is important for the predator itself, as it provides a source of nutrients^56^. Prey overkilling is not directly beneficial to the predator, but it is important in terms of protecting crops from pests. The ground dwelling spider *Pardosa agrestis* fed less on thiacloprid-treated prey than on control prey, but the rate of overkilling increased, especially among female spiders, from just 2.6% of control flies to 44.7% of thiacloprid-treated flies^55^. Similar compensation of low feeding by overkilling was also found in our results, but overkilling also decreased in comparison with control. At first sight, it seems that treatment with thiacloprid does not have a significant negative effect on pest population control, because low feeding is complemented by overkilling, and hypothetically the pressure on the pest population remains high. However, conditions in the field may differ from those in the laboratory (as already mentioned), and, together with other sublethal effects of thiacloprid treatment on the life history of predators, may reduce their long-term impact on pest populations in orchards. Due to all the above-mentioned factors, it is important to evaluate the obtained knowledge correctly and to correctly implement it when assessing the impact of thiacloprid treatment on beneficial arthropods.

### Paralysis and mortality

The toxicity of neonicotinoids to spiders is lower than to insects, most likely because the structure of acetylcholine receptors, which mediate the action of neonicotinoids in arthropods, differs between insects and spiders. In spiders, acetylcholine receptors are present^24,57^, but their sensitivity to neonicotinoids is lower than that of insect receptors^24^. We found significant mortality under thiacloprid treatment only in *Anyphaena* spiders, not in *Philodromus*. This indicates that nocturnal *Anyphaena* seems to be more sensitive to thiacloprid treatment than diurnal *Philodromus*. On the other hand, *Philodromus* suffered significant temporary paralysis caused by tarsal contact with thiacloprid. Although we observed the recovery of all affected *Philodromus* individuals within 14 days under laboratory conditions, such paralysis could be fatal in natural conditions due to the increased risk of dehydration or predation by other predators. Paralysis was observed in spiders of the family Linyphiidae under acetamiprid and thiacloprid treatment, and in *Philodromus cespitum* under treatment with acetamiprid^25^. Sýkora^58^ reported that tarsal contact with neonicotinoids (actemapirid, imidacloprid, thiacloprid and thiamethoxam) did not cause significant mortality in *Phylloneta impresa* (Koch 1881) and that no paralysis was observed. However, Řezáč et al.^24^ found that the dorsal application of thiacloprid to spiders of the family Linyphiidae caused paralysis and mortality, which was up to 57% in males and 29% in females.

Several studies have reported the paralysing effects of neonicotinoids on other non-target invertebrates, particularly honeybees. Following neonicotinoid application, bees are often found lying motionless around the hive^59^. Bumblebees showed significant growth inhibition following imidacloprid application, due to disorientation and the inability to search for food^60^. The paralyzing effects of neonicotinoids have also been found in predatory insects. For example, in the beetle *Harmonia axyridis* (Pallas 1773), 72% of larvae treated with thiamethoxam or clothianidin developed neurotoxic symptoms, including paralysis^61^.

### Other sublethal effects of neonicotinoids

There may be other sublethal effects of neonicotinoids which should be considered. For example, Korenko et al.^27^ found that after treatment with thiacloprid, male *Pardosa agrestis* (Westring 1861) are unable to complete the mating dance due to the disruption of chemical communication, which may reduce the chances of reproduction. Neonicotinoids also have a significant effect on locomotion. According to Řezáč et al.^62^, they reduce the speed of *Pardosa lugubris* (Walckenaer 1802), which may affect predation success or escape.

Further research is needed to better understand the long-term effects of neonicotinoids on spider populations and other non-target organisms. Research should focus on their persistence in soil and plants, their accumulation after repeated applications, and their interactions with other pesticides. In addition, research should go beyond predation to include sublethal effects on locomotion, food intake, and reproduction, as these factors are key to assessing the wider ecological consequences of pesticide exposure. A comprehensive examination of these parameters could provide crucial insights into the risks associated with neonicotinoid use. Regulatory frameworks should ensure that the approval of neonicotinoid-based pesticides is conditional on thorough research into their impacts on a wider range of organisms, including both lethal and sublethal effects. Prioritizing such studies over commercial considerations could help to minimise unintended ecological disturbances while maintaining effective pest management strategies.

## Data Availability

All data generated or analysed during this study are included in this published article.
